# Data-driven customer acceptance for attended home delivery

**DOI:** 10.1007/s00291-023-00712-4

**Published:** 2023-04-01

**Authors:** Charlotte Köhler, Ann Melissa Campbell, Jan Fabian Ehmke

**Affiliations:** 1 Department of Data Science and Decision Support, European University Viadrina, Frankfurt (Oder), Germany; 2 Department of Business Analytics, University of Iowa, Iowa City, USA; 3 Department of Business Decisions and Analytics, Universität Wien, Vienna, Austria

**Keywords:** Historical data, Sampling, Data-driven customer acceptance, Attended home delivery, Vehicle routing with time windows

## Abstract

Home delivery services require the attendance of the customer during delivery. Hence, retailers and customers mutually agree on a delivery time window in the booking process. However, when a customer requests a time window, it is not clear how much accepting the ongoing request significantly reduces the availability of time windows for future customers. In this paper, we explore using historical order data to manage scarce delivery capacities efficiently. We propose a sampling-based customer acceptance approach that is fed with different combinations of these data to assess the impact of the current request on route efficiency and the ability to accept future requests. We propose a data-science process to investigate the best use of historical order data in terms of recency and amount of sampling data. We identify features that help to improve the acceptance decision as well as the retailer’s revenue. We demonstrate our approach with large amounts of real historical order data from two cities served by an online grocery in Germany.

## Introduction

During recent years, there has been a rapid growth in home delivery services, including during the ongoing COVID-19 pandemic. These services require sophisticated decision support, as delivery operations are costly, and customer expectations for these services are high. Recent technological improvements allow online retailers to collect a vast amount of order data, and there is a growing interest in how to use this data in making decisions. However, research for home delivery to date has rarely used this historical order data [see e.g., Chu et al. (2021)] although data-driven models have shown their benefit for other application areas [see e.g., Zeng et al. (2021)]. We are interested specifically in how to best use this real data to manage scarce delivery capacities efficiently.

Home delivery services require the attendance of the customer during delivery, so retailers and customers mutually agree on a delivery time window in the booking process. Thus, capacity management includes decisions about the time window offerings for customers who are in the process of ordering as well as properly reserving capacity for those customers that have not yet ordered, but may significantly contribute to the retailer’s revenue. This is especially important for retailers where the demand in many time windows exceeds the capacity of their limited fleet of delivery vehicles. Since online booking processes require immediate replies to customers requesting a delivery, the amount of historical data to consider is also an important decision. We will follow the example of an online supermarket that offers time windows to customers requesting delivery during an online booking process. Since most online supermarkets are still struggling to create a viable business model, accepting customers in a way that maximizes the retailer’s revenue is very important.

Several ideas on how to support decision making for customer acceptance have been presented in the literature. Some ideas focus on myopic cost optimization. For these, a retailer would estimate the costs of accepting a request for a specific time window and would either apply static acceptance rules (Ehmke and Campbell 2014) or dynamic mechanisms based on evolving route plans (Campbell and Savelsbergh 2005) to efficiently distribute delivery capacity. Extending this, retailers could also try to influence the time window choice of a customer, e.g., through adjusting delivery fees (Campbell and Savelsbergh 2006, Klein et al. 2019) or lengths of offered delivery time windows (Köhler et al. (2020)). However, instead of simply reacting to new information, retailers can make acceptance decisions by anticipating future demand through considering patterns of historical demand (Cleophas and Ehmke 2014).

Even though all of the above work represents established customer acceptance mechanisms for attended home delivery, none of the approaches are tested solely with real data: The delivery network is often approximated (based on a mathematical formula or an exemplary road network), customer locations are drawn randomly (often based on a grid with probabilities for customer requests within each square), arrival time rates in the booking process are ignored, time window choices are assumed to be uniformly distributed or based on a simple probability function, and/or the revenue associated with an order is not considered. To the best of our knowledge, the decision on how much historical order data to consider and how to best represent features of historical order data to anticipate future demand has not been investigated in the context of customer acceptance in any of the existing academic literature. In dynamic routing, as discussed in Sect. 2.2, there are several papers that propose the use of sampling to make routing decisions. While the use of sampling is promising for use in customer acceptance decisions for attended home delivery, the work in this area also has not investigated the proper use of historical data in sampling design.

In this paper, we propose a data-science process for determining the best use of historical order data in making more informed customer acceptance decisions for a setting with a limited delivery capacity. Being the foundation of any data-driven analysis, we provide a detailed process for understanding this data before using the resulting information to inform our choices. We will focus on a sampling-based approach for making customer acceptance decisions for home delivery problems, as this is a promising solution method that is clearly heavily reliant on data. Whenever a new request arrives, routes are created that consist of already accepted customers and potential future customers based on samples derived from historical order data. These routes can support automated decision-making on whether the present request should be accepted or not, and the final routes can help us determine which of the sampling choices is most effective for a particular problem or company. Computational time available during the booking process limits the number of routing problems that can be solved, and hence the selected input data to create the samples should be chosen carefully in terms of recency and amount of data.

We demonstrate our approach with a case study of two cities served by an online supermarket in Germany. We show that working with real data adds many challenging and insightful facets to the problem of customer acceptance. For example, although sampling approaches are known to achieve close-to-optimality results with increasing sample size (Hvattum et al. 2006), we find that using more historical data to increase sample size beyond a certain point can have a negative effect on solution quality. We will discuss how we can reduce the number of samples needed to maximize revenue through more informed order acceptance based on a systematic analysis of the data input and a problem-specific combination of booking features within the samples. We compare the results from different sampling designs with results from a simple myopic approach, as commonly used by many online supermarkets. In this way, our results can demonstrate the value of using historical data via samples.

This paper is structured as follows. In the next section, we give an overview of the literature in terms of current approaches for customer acceptance for attended home deliveries and dynamic vehicle routing. In Sect. 3, we present our problem definition. Section 4 describes the creation and evaluation of samples through a sampling-based acceptance mechanism. In Sect. 5, we present a detailed data analysis for our case study. We summarize managerial insights in Sect. 6 and conclude in Sect. 7.

## Related literature

We consider a booking process in which customer requests arrive dynamically and are accepted until a specific cut-off time before a final route plan is created and executed. The challenge is that when making these acceptance decisions, future requests are unknown, and the retailer wants to use the limited fleet capacity wisely. Thus, it is hard to know which subset of customers will yield the highest profits. Customer acceptance mechanisms help the retailer with this decision. We present and distinguish these according to how they explore the information available during the booking process within Sect. 2.1. In addition, in Sect. 2.2, we will consider dynamic routing approaches that do not focus on customer acceptance but are similar to our approach in terms of the use of sampling.

### Customer acceptance

First, a large part of customer-acceptance literature considers only *real-time available demand information* in the course of the booking process. With this approach, a set of tentative route plans is maintained during the booking process, which are used to decide if a current request can be accommodated or not with given delivery capacities. This allows decisions to be made very accurately based on actual demand. However, these approaches do not take any information about future requests into account. Thus, no capacity is reserved, decision making is greedy and often far from optimal. Corresponding approaches can be found in Campbell and Savelsbergh (2006), Hungerländer et al. (2017), Cwioro et al. (2019), Köhler et al. (2020), Köhler et al. (2019), and Yang et al. (2016).

In contrast, some literature presents static acceptance rules parameterized from *aggregated historical demand information*. As a counterpart to dynamic acceptance, static acceptance rules, such as how many orders to accept in a particular time window, can better account for the total expected demand. To ensure feasibility in delivery routing, the resulting estimates are often conservative, so there is a risk that capacity will not be utilized. In Ehmke and Campbell (2014), the number of customers to accept for each time window is predefined based on historical capacity utilization and demand. In Cleophas and Ehmke (2014), expected demand is used to estimate capacities per time window and delivery area in conjunction with expected revenues. Köhler and Haferkamp (2019) investigate static acceptance for varying demand settings and show that for imbalanced demand and density of customer locations, static acceptance is inferior compared to dynamic acceptance.

Lastly, there is a line of literature that *links real-time demand information with historical demand information*. Klein et al. (2017) estimate the delivery costs of a request based on already accepted requests and expected future demand. The authors do not create estimates of future demand, but assume that a sufficient forecast is available and focus on the impact of pricing decisions for delivery options for future customers.

With sampling, potential realizations of future demand (e.g., future customer requests) are created using historical data to evaluate possible solutions (Ghiani et al. 2009). Because sampling links current and historical information, it is seemingly “better” at profitably allocating capacity. For sampling to work, the assumptions used to create these samples must be accurate. A robust solution is often sought by solving for as many different demand scenarios as possible. However, this can be quite difficult to accomplish due to the limited computation time available during the booking process. As a result, only few customer acceptance mechanisms use sampling. In Campbell and Savelsbergh (2005), whenever a new request arrives, a single profit-maximizing team orienteering problem is solved for the set of all accepted customers, the current request and potential future requests. If the current request is included in the found solution, it is accepted, reflecting that this customer is likely to be more profitable than future customers. Azi et al. (2012) test the performance of a sampling approach for an increasing number of scenarios. The authors show that increasing the number of sample problems to be solved initially increases the improvements compared to myopic solutions, but after a certain number they plateau. Both approaches are very similar to our evaluation sampling approach, in which we also create routes for a set of known and expected future requests. However, Campbell and Savelsbergh (2005) and Azi et al. (2012) both assume exogenous probabilities of future requests, while we investigate what data is suitable for creating instances that best reflect expected requests.

Although some of the presented customer acceptance mechanisms include historical demand data, very few test their approaches with real data. Ehmke and Campbell (2014) and Cleophas and Ehmke (2014) use a fictitious delivery setting inspired by the real-world road network of Stuttgart, Germany. Köhler and Haferkamp (2019) use a subset of the dataset that we use. In Campbell and Savelsbergh (2005) and Klein et al. (2017), customer requests are drawn uniformly from a grid that serves as the delivery area. Among the few to consider large amounts of historical order data are Yang et al. (2016), who also have access to a large data set from an online supermarket. However, they use the data to estimate a customer choice model, whereas we investigate the data for customer acceptance decisions. In contrast to Yang et al. (2016), we do not estimate demand but use the actual time window and delivery location combinations as they occurred in the historical order data of an online grocery.

### Dynamic routing

Unlike with customer acceptance, sampling is often investigated for dynamic routing applications in which customers arrive dynamically and are assigned to vehicles en route. Here, customer acceptance and delivery overlap (at least partially), which is often the case for service scheduling or pickups (and not applicable for the delivery of goods that first need to be picked and collected on the truck before departure). In contrast to customer time window offering decisions, the focus is not only if a request should be accepted or rejected, but also how delivery routes should be changed (i.e., if a new customer should be visited now or later). An overview of dynamic routing literature can be found in Ulmer (2020). Bent and Van Hentenryck (2004) were one of the first to present a sampling approach for customer acceptance in dynamic routing. We will use their idea as an input on how to translate sampling results into acceptance decisions in Sect. 4.3. Van Hentenryck et al. (2010) discuss the ratio of known and unknown customers during a booking process, which is known as “degree of dynamism.” In our problem, none of the requests are known at the beginning of the booking process, so based on the findings of Van Hentenryck et al. (2010), we expect sampling to outperform greedy customer acceptance. Ghiani et al. (2009) try to find the most promising problem solutions for a subset of sample scenarios to save computational time. This leads us to believe that some historical booking days can represent current order patterns better than other days. Although the literature mentioned in the above paragraph is important in the context of sampling procedures, all are again based on artificial data.

Next, we will present insights from sampling in dynamic routing literature that are related to the ideas of including real data. Hvattum et al. (2006) use sampling to decide on which customer to service next with the objective to minimize routing costs. They include real-world data about arrival patterns of bookings and delivery locations from a logistics company. Based on this data set they show that with an increasing number of samples tested, solution quality increases and approaches the optimal solution. Although Hvattum et al. (2006) are one of the few approaches that are very similar to us in the way that they include a large real-world data set in their sampling approach, there are many differences. First, their problem does not contain any time windows, whereas we consider time window choices of customers. Time windows are known to increase the complexity of routing problems and also make it much harder to assess what the future impact of accepting a customer is. Second, they separate the customer location and booking behavior information in the creation of their samples. Instead, we will explore the value of linking this information about customers in the samples. Third, Hvattum et al. (2006) increase the number of solutions created based on samples, but they do not change the input data from which the samples are created. In contrast, we will consider the interplay between the number of samples and the recency of historical order data reflected in the samples.

Lochem et al. (2020) are one of the few to investigate in detail which historical requests should be included in samples to most accurately represent upcoming demand. In a same-day setting based on a large real-world data set from a flower delivery company in the Netherlands, they first cluster historical requests to identify emerging groups of requests. In the next step, they predict the frequency for each of the groups of requests that is then used to create future requests within the samples. They do not take into account customer revenues, and the time windows are very long and hence rarely limiting (mostly 14 hours). Similar to us, the authors also consider how data can be best represented in the samples. The authors show that the greater the match between historical requests with new customer requests, the better their approach works. We address this by considering the amount of historical data that needs to be included in sampling-based customer acceptance.

## Problem description

We motivate our problem by the practice of many online retailers that manage customer requests during an online booking process. Within the booking process, customer requests arrive at booking time $t>0$ and ask for a time window offer for delivery service. We assume that the retailer knows the revenue associated with accepting that request (based on the filled shopping basket) and the delivery location (for most online supermarkets, this has to be revealed before booking a delivery). The retailer’s objective is to maximize the overall revenue *R*, and the retailer has to decide which time windows to offer to achieve this goal. For each request, the retailer offers a set of time windows with identical delivery fees and length that are feasible (i.e., in which the request can be serviced and no delivery promises of already accepted customers are violated). If the revenue is the same for each customer, this objective becomes making deliveries to as many customers as possible each day.

The retailer has a limited fleet *V*. Each vehicle $v \in V$ can provide delivery service for a total duration that corresponds with the time span of the offered time windows. The total number of accepted customers is therefore limited by the availability of the vehicles within the time windows. Accepted requests and the promised delivery time windows cannot be withdrawn or changed. Thus, the offer of a particular time window to new customer request *i* is only allowed if already accepted customers can still be guaranteed delivery within their time windows. Whenever a new request *i* arrives during the booking process at booking time *t*, immediate feedback is required as to whether this request should be accepted or rejected.

Each customer has a single time window preference. If the customer’s preferred time window is included in the time windows the retailer offers, the delivery is confirmed. If not, the customer cancels the booking process. (A customer could also choose a different time window, but we initially only consider a customer’s single time window preference.) Requests are considered for acceptance until a specific cut-off time *T* that is before the day of order delivery, i.e., on-demand deliveries are not considered within this paper.

The retailer saves records of all historical requests. For each of these requests, the retailer knows the following features:

- The *booking time* *t* during the booking process at which a customer requested the delivery,
- The *delivery location* for the order,
- The *delivery date* associated with the particular booking process,
- The *time window* that was confirmed for delivery,
- The *revenue* *r* that is associated with accepting the order, which can either represent a monetary value (e.g., the basket value) or a non-monetary value (e.g., the customer status).

One of the well-known challenges associated with this problem is the impact of the acceptance of early requests on the ability to accept later requests. This is important if later requests cause fewer detours in the route plan or have higher revenues associated with them. The approach adopted by other authors such as Campbell and Savelsbergh (2005) is to make acceptance decisions using sampling. If accepting request *i* at time *t* for delivery during a particular set of time windows is generally considered to be beneficial given a set of samples of future requests, then the time window is offered (the customer is only offered feasible *and* beneficial time windows). To create the required samples, we consider the available historical data and investigate how to best use the data to represent potential future bookings in the current booking process.

## Methodology

We present our methodology to determine the use of historical order data to create samples for data-driven customer acceptance. We divide our procedure into three steps: *understanding the data*, *sample creation*, and *sample evaluation*. In Sect. 4.1, we detail the methodology required to examine large amounts of historical order data. In Sect. 4.2, we present our sampling approach for different representations of historical order data and describe how we identify the best configuration through sample evaluation in Sect. 4.3. While a data-driven approach needs to be tied to the data set at hand, our methodology is quite generic and allows for transferring of our findings to any online retailer that stores historical order data.

### Understanding the data

To gain a general understanding of historical order data, we follow a data-science approach as proposed in Provost and Fawcett (2013). The core assumption is that based on this data, we can extract meaningful information for future booking processes. To investigate the value of historical order data in customer acceptance, we first conduct an exploratory analysis of the data set. The goal of exploratory data analysis is a profound understanding of the data (Hand et al. 2001).

For our visual exploratory data analysis, we apply the process proposed by Keim (2002). This involves understanding how much data is available and how the data is structured. Thus, we will discuss the type of data typically available in historical order data for attended home delivery. Each order consists of *variables* that contain information about the five features as described in Sect. 3. Each of these variables is of a particular data type: *categorical variables* have a limited set of possible outcomes that do not necessarily correspond to an ordering, whereas *numerical variables* are not limited and can be ordered (Van Der Aalst 2012). For the five features considered, we expect categorical data on the set of time window options and preferred time windows; however, we assume that most variables in the data set are numerical, such as delivery date, booking time, revenue, and location data. As historical order data has usually been collected over some time in terms of sequential observations, they belong to the group of time series data (Esling and Agon 2012). Therefore, to describe our data set, we will use an *index* for each order corresponding to a particular delivery date. All orders associated with a particular delivery date represent an instance.

We can use visualization techniques to learn more about the characteristics of the historical order data.

- We can investigate if there are differences in the amount and types of orders corresponding to delivery on different days of the week. This can guide whether the historical order data used to help to make decisions on a particular day should come more from recent orders from any day or should be restricted to the same day of the week.
- Similarly, we can understand the distribution of time window choices among the different delivery time windows.
- Last, it is also helpful to analyze locations of the orders in a particular data set, which helps to create an understanding of the layout of the delivery area and the order density in different parts of the city.

These efforts are important because time windows and days with the largest numbers of orders and areas with high-order density are the most critical ones for capacity management and hence acceptance decisions.

### Creating data samples

Once we have a good understanding of the historical order data, we need to investigate which of the available data best generalizes previous booking behavior so we can properly generate samples for our sampling-based prediction of potential future requests. To this end, we consider how much historical data should be included and derive information on the historical booking processes from the combination of features as well as from different ways of aggregating historical data.

First, historical order data may have changed over time and may not reflect current customer behavior appropriately, so that older data no longer gives an up-to-date picture (*recency* of the data). For example, in the case of online supermarkets, initial order data may contain many customers who have tried the service once but are not returning. Furthermore, the online supermarket’s offering could have changed over time by adjusting the offered delivery time windows. Seasonal shopping behavior may be another reason for varying ordering behavior captured in the data, e.g., more expensive purchases in the winter season. The more change we see in the historical data over time, the more carefully we need to define the horizon *D* of historical order data to consider in the creation of our samples.

Second, for a given horizon *D* of historical order data, we need to decide how the data should be represented in the samples to support decision making for previously unseen booking processes. Different ways of aggregating historical order data may be used. In the following, we describe the different ideas on using the historical order features:

- When assessing a new request in the light of historical order data, we can use exact instances of booking processes from a day in the booking history. We will call this approach *instance-based sampling*. This would be the most accurate representation of historical order data, but may not generalize well to predict future orders for this reason. This would also mean that the retailer would have to store all of the data for the *D* past booking processes in order to use them for the samples.
- It is possible to consider historical orders by sampling from different booking days. We will call this approach *order-based sampling*. With this approach, we would need to determine the number of orders for each sample based on the distribution of the number of orders over the specified horizon *D*. This can be advantageous if the historical data contains exceptions of significantly more or significantly fewer orders, e.g., due to a holiday. However, we would possibly lose booking process-specific information by combining the orders, as customers might always order on a regular basis and thus the same customers may be included multiple times in the same booking process.
- Third, following the standard of related sampling literature, we could derive samples from distributions of individual features or consider the most correlated ones in joint distributions of historical order data. We will refer to this as *distribution-based sampling*. These might be continuous distributions, such as for booking time, or discrete ones, such as for time windows. We will investigate which of the features are correlated to decide where individual distributions or joint distributions of the features are needed.

The way we create our information also affects how many samples we can generate. For instance-based sampling, the number of samples that can be considered is limited, but we can create a large number of samples for order-based and distribution-based sampling. Again, understanding the data is an important first step to decide whether we should include the historical order data in as much detail as possible in the samples or whether aggregating or combining the data will provide a better prediction of potential future requests.

### Sample evaluation

We propose a sampling-based acceptance mechanism for delivery routing that dynamically builds route plans to determine the feasibility of a new request based on the current booking process. This mechanism will be used to evaluate different choices in the use of historical order data to create samples.

For a given horizon *D*, we generate *S* samples for each request *i* derived from the historical data that we use to decide if we want to accept or reject the current request. Each sample contains information about all previously observed first-choice requests, the current request *i*, and a set of potential future requests created according to instance-based, order-based, or distribution-based representation to reflect potentially upcoming customers.

For each sample, we create route plans. In particular, we solve a team-orienteering routing problem with the objective to maximize the overall revenue given the available fleet capacity. Contrasting standard routing problems, team orienteering identifies the most beneficial subset of customers in terms of total revenue *R* in the route plan (Vansteenwegen et al. 2009). This results in a route plan $P_s$ containing a route for each of the vehicles in which all already accepted requests as well as the most valuable potential future requests (from the sample) and/or the current request are included.

Having created a set of routes based on samples, we need to make a decision on whether to accept the current request or not. For dynamic routing problems, it is very common to apply a consensus function that seeks similarities in obtained solutions (Pillac et al. 2013). In the context of dynamic routing, this simply means that the request that appears most frequently in the sampled solutions will be visited next. In the context of customer acceptance, we will be able to change the order of customer visits and vehicle assignments in the course of the booking process, though. Hence, it can be an advantage to consider not only the frequency, but also the robustness of an acceptance decision (Bent and Van Hentenryck 2004), which corresponds to the desired level of generalization from historical bookings. We apply the consensus idea and measure how often a request *i* is included within all route plans created from samples and solved with an acceptance limit $\theta$. This acceptance limit indicates the minimum number of route plans a new request has to be included for it to be accepted.

For applying this acceptance criterion, we need to decide on the value of $\theta$ as well as how many samples *S* we want to consider. In the related sampling literature, the guiding principle is often “the more the better” (as shown by Hvattum et al. (2006)). However, since solving more samples takes more time (which is hardly available in an online booking process) as well as more data storage and processing efforts, the number of samples for an online retailer should be as small as possible. For both cities in our case study, we will therefore analyze the appropriate time horizon as well as the number of samples needed in Sect. 5.4.

The retailer needs to identify the best configuration for the sampling procedure. Hence, in the following, we will systematically test and compare different configurations. The best configuration will be identified following common data-science standards. To this end, we will divide the historical order data into training and test data. The training data is used to form the input data for the three sampling variants as described above and will hence guide customer acceptance decisions. The test data will be used to create “actual” (simulated) booking processes of requests not used for training and will serve to evaluate the performance of a sampling procedure. Depending on the nature of the data, we expect some configurations to work better for some of the simulated booking processes than for others. However, our goal is to find a configuration that generalizes well, i.e., that is suitable for new, previously unseen requests. Therefore, we will determine the best configuration for the sampling procedure based on the average of the results of all simulated booking processes for two cities.

We compare the results of sampling-based prediction of customer acceptance with two benchmarks that we call *hindsight* and *myopic*. For the hindsight benchmark, we assume that we know all requests at the beginning of the booking process and can therefore choose the most profitable subset of requests to be served, i.e., we have perfect information on future customers at all times. This obviously would not be possible in reality but represents an upper bound for the total revenue in our experiments. For the myopic approach, we accept customers in a greedy manner without considering any information on future customers. This means that every customer is accepted as long as capacity is available. This strategy has been implemented by many online supermarkets.

## Case study

In this chapter, we investigate data-driven customer acceptance based on a large data set of historical order data provided by the German online grocery AllyouneedFresh. We will first investigate data understanding and exploratory data analysis of our data set (Sect. 5.1). Then, we will examine how we create the different sampling variants from historical data (Sect. 5.2). Finally, we will demonstrate how the different sampling approaches are evaluated (Sect. 5.3) and analyze which representation of historical order data is best for data-driven customer acceptance in our case study of two German cities (Sect. 5.4).

### Understanding the data

AllyouneedFresh provided historical order data for the period from September 2016 to May 2017. At the time of data collection, AllyouneedFresh was a subsidiary of DHL and could therefore rely on the large DHL fleet for order delivery. Therefore, customers’ preferred time windows could always be accepted. For this study, we use this data but consider a fleet of two delivery vehicles. This allows us to accept all requests on days with very few orders, but we have to reject customers on most days, especially in high demand time windows and assume that customers whose preferred time window is not available will leave the booking process.

Customers could choose from a variety of groceries on the AllyouneedFresh website and had them delivered to their homes. All over Germany, customers could pick a delivery on the day of their choice. In addition, customers in 14 metropolitan regions could choose from a selection of two-hour delivery time windows. AllyouneedFresh offered six consecutive time windows, with the first time window starting at 10:00 in the morning and the last time window ending at 22:00. In addition, there was another overlapping two-hour time window from 19:00-21:00. Each order is described through the time of booking, the selected delivery day as well as the selected time window, the delivery address, and the value of the shopping basket that we will use as estimation of the retailer’s revenue.

We analyze the historical order data first concerning distribution over weekdays. Some weekdays are more popular than others, with about a fifth of all orders delivered on Fridays. Tuesday, Wednesday, and Thursday have a share of 18% of deliveries each. The number of deliveries is generally lower on Saturdays (14%) and the lowest on Mondays (11%). Since there is such variability among weekdays, we focus on those bookings that were delivered on Fridays for our study.

In our case study, the first Friday will be September 30, 2016, and will be indexed as *Friday 30*. The latest Friday will be June 30, 2017, and will be indexed as *Friday 1*. All Fridays in between will be indexed accordingly. Note that in some weeks no deliveries were made because of a public holiday. Since most online supermarkets are offering their service in metropolitan areas, we will choose two cities out of the data set from different regions in Germany with a comparable number of orders but no overlapping customers.

**Fig. 1:**
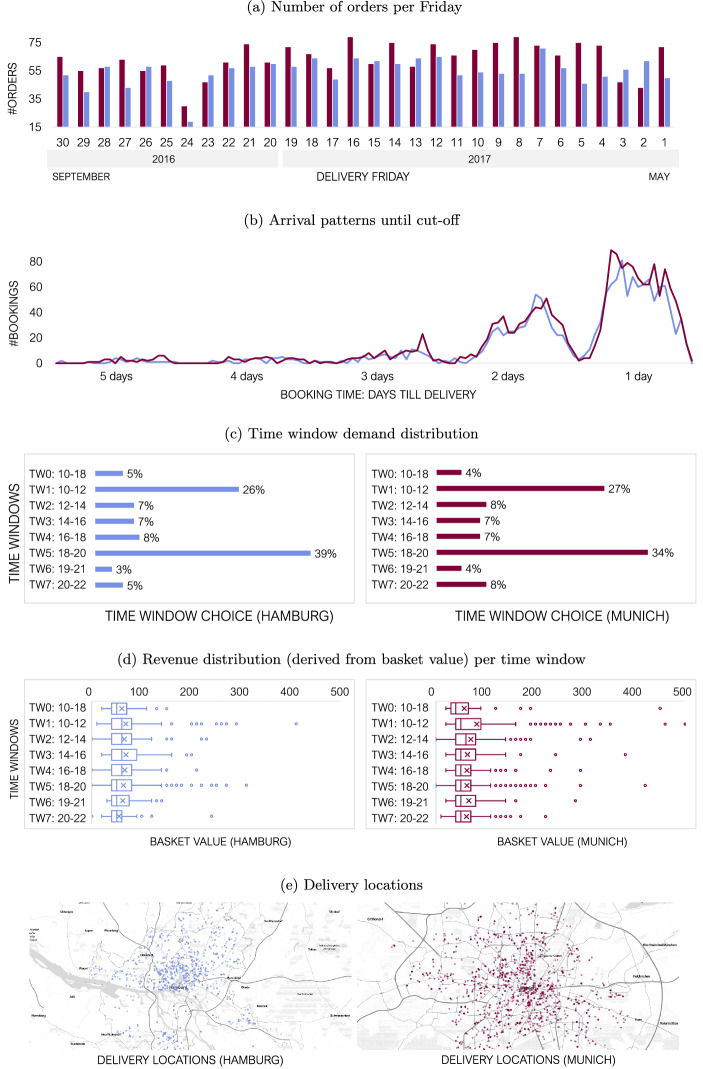
Visualization of historical order data for deliveries on Fridays in Hamburg and Munich, September 2016–May 2017

The first city is Munich, which is located in southern Germany, and the second city is Hamburg, which is located in the northern part of Germany. In Fig. 1, we provide visualizations of the order data characteristics for Hamburg (blue) and Munich (red) for all bookings with deliveries on Fridays. In Fig. 1a, the number of deliveries on these Fridays is shown. For Hamburg, we have about 54 orders for each Friday, booked an average of 29 hours before cut-off, with an average revenue of about €65. For Munich, we have about 64 orders for each Friday, booked an average of 28 hours before cut-off, with average revenue of about €68. Table 10 in the Appendix 8.1 presents detailed statistics for each of the Fridays. While the total number of deliveries is comparable between Hamburg and Munich, there are some Fridays which show a large difference, e.g., Fridays 4 and 8. For both cities, Friday 24 stands out because of a significantly lower number of deliveries. It will be interesting to see how sampling performs for this day or for other days using orders from this low demand day. In the following, we investigate how historical order data collected on past Fridays can help to improve customer acceptance on future Fridays.

In Fig. 1b, we focus on when bookings were made relative to the cut-off time. To this end, we plot the number of orders per hour prior to delivery. The *y*-axis reflects the number of orders per hour and the *x*-axis represents a timeline with the cut-off time $T=0$ being on the very far right. The booking behavior seems to be surprisingly similar for Hamburg and Munich. We can see that there are fewer bookings during the night hours and more bookings during the day. Furthermore, only a few customers order early, i.e., five to three days before delivery. Instead, many customers order within 48 hours before delivery, and most customers order within 24 hours before delivery, which underlines the importance of taking the time of booking into account within sampling during this time period.

Next, we characterize the preferred time window choice observed in our historical order data (see Fig. 1c). On the *y*-axis, we depict the eight available time window options. In general, demand for time windows in both cities is very similar with a slightly higher demand for the very late time window from 20:00-22:00 in Munich than in Hamburg. Two time windows are in high demand: 10:00-12:00 as well as 18:00-20:00. Following the same line of visualization, Fig. 1d indicates the distributions of basket values in the different time windows, indicating the chance for higher revenues in the most popular time windows.

Finally, in Fig. 1e, the spatial distributions of all delivery locations of all Friday bookings are plotted on a map. Each dot represents a customer address; darker dots represent multiple deliveries to the same address. For both cities, we can see a more dense distribution of deliveries made in the inner city than in the outskirts. However, the city of Hamburg is characterized by many canals and a large port, leaving a gap in delivery addresses in the southern part of Hamburg. Such a graph highlights the importance of using real data in sampling rather than uniform distributions over a rectangle, for example. This suggests the use of sampling from actual locations even for distribution-based sampling. This should keep the geographical spread of locations consistent with city-specific patterns.

We noticed that (1) the number of orders per delivery day varies greatly, (2) most of the customers request as early as one day before cut-off, (3) demand for time windows is high, especially for early morning time window TW1 and early evening time window TW5, and (4) customers with a high revenue request these time windows. Demand is also distributed with city-specific patterns and should be sampled to reflect these.

### Creating data samples

Given our understanding of the order data from the two cities, we now discuss the details of instance-based, order-based, and distribution-based sampling.

To this end, we must define the horizon D for the source of the historical order data. The horizon is determined as in Sect. 5.3, but is used to define the set of samples for evaluation as follows. A visual description of these ideas is included in Table 1. Note that all orders considered below are first-choice orders.

**Table 1:** Creating data samples from the historical data

Sample generation	Booking time	Revenue	Time window	Delivery location	#Orders
Instance-based	Historical orders from one instance in horizon				
Order-based	Historical orders from different instances in horizon				Distribution
Distribution-based	Distribution	Distribution	Distribution	Distribution	Distribution

- For instance-based sampling, we preserve all historical orders as seen in an instance from a historical booking day within the horizon. Each sample represents an exact mapping of a historical booking day from the horizon, including all orders and their features.
- For order-based sampling, we again preserve all features that belong to a historical order, but we combine the orders across historical booking days from the horizon. To this end, we average the number of orders across the horizon and compute the variance. Based on this, we randomly pick the number of orders for a new sample and sort the orders according to the time of booking.
- For distribution-based sampling, we need first to learn if there are dependencies between historical bookings and their features. In Tables 2 and 3, we show the calculated correlations for the order features for Munich and Hamburg, respectively. Latitude and longitude are derived from the location feature. Recall, further features include time window, revenue, and booking time.
  For both cities, there is a correlation between the selected time window and booking time, which could imply that a combined consideration of these two features in the samples is important. Surprisingly, there is no strong correlation between the location feature and the remaining features. However, there is a correlation between latitude and longitude, which can be explained by the nature of the city network. There is very little correlation between location and time window or value, indicating that there are no significant differences in residential areas and booking behavior. Another surprising result is that the revenue cannot be derived from the delivery location, time window choice, or booking time. This means that it is probably more difficult to anticipate whether requests with high revenues are still to be expected and how much capacity should be reserved for them. Following the results from the correlation analysis, we will explore two variants for distribution-based sampling:
  - *Distribution-based*$_{C}$*:* With the first variant, we consider the correlation between time window and booking time and preserve these features in the samples. In particular, we pick a random number (based on the distribution over the horizon) to determine the number of potential requests in the samples as in *order-based* sampling. For each request, we pull a combination of the features of time window and booking time from the historical order data from the horizon to reflect their correlation but choose the location and value features from discrete distributions based on data over the horizon.
  - *Distribution-based*$_{R}$*:* Since the correlation between time window and booking time was not particularly strong (under 0.5), we use randomly sampled features based on distributions with this variant. In particular, we create samples as in *distribution-based*$_{C}$, but determine time window choice and booking time based on an empirical distribution derived from the historical order data.

**Table 2:** Feature correlation for Munich

**Lat**	−0.11	−0.06	+0.01	+0.01
	**Lon**	−0.04	−0.03	−0.04
		**TW**	−0.03	−0.25
			**Revenue**	−0.02
				**Time**

**Table 3:** Feature correlation for Hamburg

**Lat**	+0.17	+0.10	+0.09	+0.04
	**Lon**	−0.02	+0.09	−0.02
		**TW**	−0.04	−0.22
			**Revenue**	+0.01
				**Time**

### Evaluating the samples

Our evaluation of historical order data is based on the simulation of order processes and the solution of the involved routing problems. To this end, we use *Google OR tools* (Perron and Furnon 2019) and embed them in a customer-acceptance simulation. To create a routing solution, Google OR tools perform a construction and improvement heuristic. For the construction step, we let Google OR tools automatically choose a solution strategy (e.g., savings or sweep); the improvement step is a guided local search. We set the solution time for each run of the routing algorithm to 10 seconds. To increase the quality of the solution, we save the best routing solution found after a customer has been accepted and use it as the initial solution for the next customer.

We assume a service time of 12 minutes which includes parking and handing over the order at each customer (as in Köhler et al. (2020)). We use a fleet of two delivery vehicles. This allows us to accept all requests on days with very few orders, but we have to reject customers on most days especially in high demand time windows. We use travel time data provided by GraphHopper Directions API^1^ to compute routes. The mean travel time between all possible delivery locations is 17.0 minutes for Hamburg and 11.6 minutes for Munich. We assume a working day of 12 hours, parallel to the beginning and end of the first and last time windows at 10:00 and 22:00. We are neglecting working time regulations.

To identify the best sampling procedure, we consider all booking processes with deliveries on Fridays, indexed from a past Friday (30) to the most recent Friday (1). For easier reading, we will simply call them “Fridays” from now on. For both cities, we select the ten most recent Fridays (Fridays 1–10) as test data for our simulation. Each request is considered at the time it actually arrived with exactly the same characteristics (revenue, delivery location, and time window, 8-hour or one of the 2-hour time windows). Then, for each of these ten test Fridays, we perform sampling-based customer acceptance based on the preceding 20 training Fridays derived according to the particular procedure (instance-based, order-based, or distribution-based sampling). We analyze the performance of the sampling procedures and vary the amount of historical data used as input for sample creation, i.e., the number of Fridays considered.

We will vary the length of the horizon for instance-based sampling to assess the recency of the historical order data between $D = 4$ to $D = 20$ weeks. With the most effective horizon, for the different sampling procedures, we will vary the number of samples that will be used to create the required acceptance information. We begin with a few samples, $S=4$, and will increase the number of samples up to $S=20$. Note that in an online booking process, there may not be sufficient time to process a large number of samples. Based on preliminary experiments, we set the acceptance rule ensuring that a request must be included in at least half of the sampled solutions to be accepted, i.e., $\theta =\frac{\vert S \vert }{2}$. These experiments demonstrated that a very high limit results in a lack of customer acceptance. Lower acceptance limits enable us to accept more consumers, but we were unable to produce better outcomes than with $\theta =\frac{\vert S \vert }{2}$. Appendix 8.3 contains the corresponding experiments.

### Computational results

We will discuss the most effective horizon for sampling-based customer acceptance in Sect. 5.4.1. We will identify the best sampling procedure given different numbers of samples in Sect. 5.4.2. All results in Sects. 5.4.1 and 5.4.2 will be evaluated relative to myopic and hindsight benchmarks. We present the benchmark results in Tables 4 and 5 for the ten test Fridays. For Munich, on eight out of ten Fridays, the myopic strategy collects less revenue than hindsight. For Hamburg, the collected revenue is generally smaller due to the slightly lower number of orders. With very few exceptions, for both cities, requests that were rejected in solutions were those that requested the highly demanded time windows TW1 or TW5. An illustrative example detailing how the booking processes evolves when applying myopic or hindsight can be found in the Appendix (see Sect. 8.2).

**Table 4:** Results for hindsight and myopic for Hamburg (total revenue)

	Friday									
	10	9	8	7	6	5	4	3	2	1
Hindsight	3280	3180	3330	3370	2900	2920	3350	3260	3600	3010
Myopic	2990	3020	2870	2480	2470	2740	3170	2990	3300	2470
Gap	8.8%	5.0%	13.8%	26.4%	14.8%	6.2%	5.4%	8.3%	8.3%	17.9%

**Table 5:** Results for hindsight and myopic for Munich (total revenue)

	Friday									
	10	9	8	7	6	5	4	3	2	1
Hindsight	4300	4400	4200	4320	5120	4850	4530	3920	2570	3970
Myopic	3860	4050	3410	3790	4580	4190	3970	3920	2570	3620
Gap	10.2%	8.0%	18.8%	12.3%	10.5%	13.6%	12.4%	0.0%	0.0%	8.8%

#### Recency of data

We first investigate the most-effective order horizon using instance-based sampling. In Table 6 and Table 7, we present the results of instance-based sampling for Munich and Hamburg. For each table, the results are compared to the revenue of the myopic (*M*) and the hindsight (*H*) solution in percentages, computed as (Sampling$-M$)/*M* and (Sampling$-H$)/*H*, respectively. Each column indicates for which Friday the result was obtained, and each row represents a different value for *D*. Note that for instance-based sampling, using more Fridays also means considering more samples in the acceptance decision. The rightmost column shows the average of sampling results for all tested Fridays in each table.

**Table 6:** Sampling results in relation to hindsight (H) and myopic (M) in percentages (%) for Hamburg and different horizons *D*

			Friday 10		Friday 9		Friday 8		Friday 7		Friday 6		Friday 5		Friday 4		Friday 3		Friday 2		Friday 1		Avg	
			H	M	H	M	H	M	H	M	H	M	H	M	H	M	H	M	H	M	H	M	H	M
Instance-Based	$\vert D \vert = \vert S \vert$	4	−3.4	6.0	−0.6	4.6	−5.7	9.4	−14.2	16.5	−8.3	7.7	−4.5	1.8	**−5.1**	**0.3**	−5.8	2.7	−7.2	1.2	**−4.3**	**16.6**	−5.9	6.7
		8	−3.0	6.4	−0.3	5.0	**−5.1**	**10.1**	**−8.3**	**24.6**	**−4.8**	**11.7**	−0.7	5.8	−6.0	−0.6	−2.1	6.7	−3.9	4.8	−5.3	15.4	**−4.0**	**9.0**
		12	−1.8	7.7	−0.3	5.0	−6.0	9.1	−8.6	24.2	−6.6	9.7	**−0.3**	**6.2**	−6.0	−0.6	−2.1	6.7	−3.9	4.8	−7.0	13.4	−4.3	8.6
		16	**−1.2**	**8.4**	**0.0**	**5.3**	−6.9	8.0	−11.6	20.2	−7.9	8.1	−0.7	5.8	−9.3	−4.1	**−1.8**	**7.0**	**−3.6**	**5.2**	−7.0	13.4	−5.0	7.7
		20	−2.1	7.4	−0.3	5.0	−7.5	7.3	−12.5	19.0	−7.6	8.5	−1.7	4.7	−7.5	−2.2	−3.4	5.4	−3.6	5.2	−6.6	13.8	−5.3	7.4

**Table 7:** Sampling results in relation to hindsight (H) and myopic (M) in percentages (%) for Munich and different horizons *D*

			Friday 10		Friday 9		Friday 8		Friday 7		Friday 6		Friday 5		Friday 4		Friday 3		Friday 2		Friday 1		Avg	
			H	M	H	M	H	M	H	M	H	M	H	M	H	M	H	M	H	M	H	M	H	M
Instance-Based	$\vert D \vert$ = $\vert S \vert$	4	−6.0	4.7	−2.7	5.7	−15.0	4.7	−3.2	10.3	−6.4	4.6	−4.1	11.0	**−3.3**	**10.3**	**−0.8**	**−0.8**	**0.0**	**0.0**	−4.8	4.4	−4.6	5.5
		8	−6.0	4.7	−3.0	5.4	**−7.1**	**14.4**	**−2.1**	**11.6**	−5.5	5.7	−4.7	10.3	−5.3	8.1	−0.8	−0.8	0.0	0.0	−5.8	3.3	**−4.0**	**6.3**
		12	−6.0	4.7	−2.7	5.7	−9.3	11.7	−3.2	10.3	−5.5	5.7	**−3.7**	**11.5**	−6.6	6.5	−1.8	−1.8	−0.8	−0.8	−4.5	4.7	−4.4	5.8
		16	−5.1	5.7	**−1.4**	**7.2**	−11.2	9.4	−3.5	10.0	−5.5	5.7	−5.8	9.1	−6.6	6.5	−2.8	−2.8	−0.8	−0.8	−4.3	5.0	−4.7	5.5
		20	**−4.9**	**6.0**	−1.6	6.9	−9.3	11.7	−3.0	10.6	**−4.5**	**6.8**	−5.6	9.3	−6.6	6.5	−2.8	−2.8	−0.8	−0.8	**−3.0**	**6.4**	−4.2	6.1

Overall, it can be seen that regardless of how long the horizon is, on average, instance-based sampling always generates a higher revenue for the retailer than a myopic strategy. There are only two Fridays where this does not apply: Fridays 2 and 3 in Munich, which do not require sampling because of low demand. Even though sampling generally generates higher revenues, the results vary greatly. We are mainly interested in how extending the horizon influences the quality of the results. In Table 7, we can see that by considering data from $\vert D \vert =4$ Fridays, an average of 5.5% higher revenue can be collected than with myopic, and 6.3% if data from $\vert D \vert =8$ Fridays is considered. We can also see that with an increasing number of Fridays, the consideration of more input data seems to have a negative effect. This is particularly evident for Friday 4: If we consider historical data of the past month, we can increase the revenue by 10.3%. However, extending this to the previous five months, i.e., the last 20 Fridays, reduces generalization capabilities reflected by a smaller revenue increase of only 6.5%.

Results follow similar patterns for the Hamburg data. In Table 6, we see that using data from $\vert D \vert =8$ Fridays increases the revenue by 9.0% relative to myopic. Again, with further increasing number of Fridays, less revenue is collected. Noticeable in the results is Friday 4. We have learned from Munich data that sampling is not beneficial on Fridays with significantly lower demand. However, Friday 4 has even more bookings than Friday 5, for which sampling achieves good results. If we look at the booking process of Friday 4 in detail, we can see that many customers ask for delivery in TW1 and TW5 as expected. However, no customers arrive in the last 12 hours before cut-off, whereas we had assumed that most customers order in the last hours before cut-off (see Fig. 1b). Hence, Friday 4 is special in the sense that customers booked earlier than usual. Consequently, sampling reserves capacity for the late customers observed in the historical order data. The results show surprisingly similar patterns. Generally, instance-based sampling works well for Munich and Hamburg, and it works best with data from eight historical Fridays.

To understand why shorter time horizons lead to better results, we want to investigate the changes of customers’ shopping behavior within the considered data. To this end, we perform a change point analysis using the *cpt*^2^ package in R and binary segmentation as a detection method for the order numbers in Hamburg and Munich. The idea of a change point analysis is to find states in which data can be grouped to the same trend and change points that describe transition into another state (Aminikhanghahi and Cook 2017). In Fig. 3, we depict the number of orders on the y-axis and the considered delivery Fridays on the x-axis (with Friday 1-10 the ones we use to make acceptance decisions). For both cities the analysis results in five detected change points (Friday 25, 24, 12, 8, and 6 for Hamburg, Fridays 25, 23, 14, 13, and 4 for Munich). Hence, five states are detected which we depict with horizontal lines in the figures. In addition, we mark the seasons with vertical lines, and we see that our data includes some fall weeks, the entire winter, and spring. This gives us the following insights. First, demand at AllyouneedFresh over the 30 weeks has many different states and changes quickly. Second, the changes in the states go almost perfectly with the change in the seasons. Thus, a trade-off has to be found between considering more data (as this usually improves the variability and thus the robustness of the decisions) and timeliness (as the data changes quickly). In our example, this trade-off is best found at $\vert D\vert =8$ on average. Please note that in our case not only the number of orders is of interest but also when customer requests arrive in the booking process, how much customers order, which time window is promised, and finally a routing decision about whether to include the customer in a delivery tour. Hence, full explainability based on analyzing data is hard to achieve, but we derive some intuition about why using samples from longer time periods might not be advantageous here.

*Insights of data recency assessment: * We have used historical order data in a sampling-based customer acceptance approach. Consistent with results in the literature, we have shown the superiority of instance-based sampling over a myopic approach. For the historical order data of Hamburg and Munich, on average, sampling is most effective with a horizon of $\vert D \vert = 8$.

#### Choosing the best sampling procedure

In the previous section, with instance-based sampling, we showed that less data resulted in the best average results for increasing the provider’s revenue compared to myopic. With instance-based sampling, we achieved an improvement of 6.3% and 9.0% for Munich and Hamburg when choosing a horizon of eight weeks of most recent booking data. In this section, we want to investigate how the results change if we keep the horizon constant, but use more or less samples and also combine the features of historical orders in our samples with order-based and distribution-based sampling. Following the best results from the previous section, we fix the amount of historical data to eight weeks and create either eight or 20 samples out of this data. Since *order-based* and *distribution-based sampling* include random elements, we repeat this process 10 times and present the average results of 10 runs. In Tables 8 and 9 , we present the results of order-based and distribution-based sampling for the 10 test Fridays for Munich and Hamburg for a given horizon of $\vert D \vert = 8$. Again, for each table, we present the relative result of sampling-based customer acceptance compared to the revenue of the myopic (*M*) and the hindsight (*H*) solution in percentages. Each column indicates for which Friday the result was obtained, and each row indicates how many samples $\vert S \vert$ per request were considered for the given result.

**Table 8:** Sampling results for different numbers of samples $\vert S \vert$ in relation to hindsight (H) and myopic (M) in percentages (%) for Hamburg and $\vert D \vert$ = 8 weeks of data

			Friday 10		Friday 9		Friday 8		Friday 7		Friday 6		Friday 5		Friday 4		Friday 3		Friday 2		Friday 1		Avg	
			H	M	H	M	H	M	H	M	H	M	H	M	H	M	H	M	H	M	H	M	H	M
Instance-Based	$\vert S \vert$	8	**−3.0**	**6.4**	**−0.3**	**5.0**	**−5.1**	**10.1**	**−8.3**	**24.6**	**−4.8**	**11.7**	**−0.7**	**5.8**	**−6.0**	**−0.6**	**−2.1**	**6.7**	**−3.9**	**4.8**	**−5.3**	**15.4**	**−4.0**	**9.0**
Order−Based	$\vert S \vert$	8	−3.9	5.4	−1.2	4.0	−6.7	8.3	−9.7	22.7	−7.9	8.2	−2.3	4.1	−7.0	−1.7	−2.8	6.0	−4.9	3.8	−5.9	14.7	−5.2	7.6
		20	−3.8	5.5	−0.7	4.6	−7.0	7.9	−10.4	21.7	−8.8	7.1	−3.0	3.4	−8.2	−3.0	−3.7	5.0	−4.9	3.8	−6.3	14.1	−5.8	7.0
Distribution-Based$_C$	$\vert S \vert$	8	−3.8	5.5	−0.8	4.5	−7.0	7.9	−11.4	20.4	−9.5	6.3	−2.5	3.9	−7.7	−2.4	−3.0	5.7	−4.7	4.0	−5.5	15.2	−5.6	7.1
		20	−3.4	6.0	−0.8	4.5	−6.6	8.4	−10.8	21.2	−9.0	6.9	−3.2	3.1	−9.1	−3.9	−3.3	5.4	−4.7	3.9	−6.4	14.1	−5.7	7.0
Distribution-Based$_R$	$\vert S \vert$	8	−3.5	5.9	−0.8	4.4	−7.7	7.1	−11.1	20.7	−9.4	6.4	−2.7	3.7	−6.9	−1.7	−3.9	4.7	−5.2	3.4	−5.7	15.0	−5.7	7.0
		20	−3.4	6.0	−0.5	4.8	−8.4	6.3	−11.4	20.4	−9.8	5.8	−2.8	3.6	−8.1	−2.9	−3.8	4.8	−4.9	3.8	−6.8	13.6	−6.0	6.6

**Table 9:** Sampling results for different numbers of samples $\vert S \vert$ in relation to hindsight (H) and myopic (M) in percentages (%) for Munich and $\vert D \vert$=8 weeks of data

			Friday 10		Friday 9		Friday 8		Friday 7		Friday 6		Friday 5		Friday 4		Friday 3		Friday 2		Friday 1		Avg	
			H	M	H	M	H	M	H	M	H	M	H	M	H	M	H	M	H	M	H	M	H	M
Instance-Based	$\vert S \vert$	8	−6.0	4.7	−3.0	5.4	**−7.1**	**14.4**	**−2.1**	**11.6**	−5.5	5.7	−4.7	10.3	**−5.3**	**8.1**	**−0.8**	**−0.8**	**0.0**	**0.0**	−5.8	3.3	**−4.0**	**6.3**
Order−Based	$\vert S \vert$	8	−6.5	4.1	**−2.6**	**5.8**	−11.2	9.3	−3.5	10.0	**−4.8**	**6.4**	−4.2	10.8	−7.5	5.5	−1.2	−1.2	−0.2	0.2	**−4.3**	**5.0**	−4.6	5.6
		20	−7.1	3.5	−2.9	5.5	−9.0	12.1	−4.1	9.4	−6,3	4.8	**−4.0**	**11.1**	−7.7	5.3	−0.9	−0.9	−0.8	−0.8	−4.7	4.5	−4.7	5.5
Distribution-Based$_C$	$\vert S \vert$	8	−6.5	5.5	−3.4	5.0	−10.6	10.1	−4.5	8.9	−5.8	5.3	−5.7	9.2	−6.0	7.2	−0.9	−0.9	−1.7	−1.7	−4.7	4.6	−4.8	5.3
		20	**−4.7**	**6.2**	−4.3	4.0	−10.6	10.2	−4.7	8.7	−6.1	5.0	−5.2	9.8	−8.4	4.5	−1.8	−1.8	−0.8	−0.8	−4.8	4.5	−5.1	5.0
Distribution-Based$_R$	$\vert S \vert$	8	−6.0	4.7	−4.0	4.3	−10.6	10.1	−4.4	9.0	−5.6	5.6	−5.6	9.3	−5.9	7.4	−1.0	−1.0	−1.8	1.8	−4.6	4.7	−4.9	5.2
		20	−6.4	4.3	−3.9	4.4	−9.5	11.5	−4.8	8.5	−6.3	4.7	−5.7	9.2	−6.5	6.7	−0.9	−0.9	−1.0	1.0	−5.1	4.1	−4.9	5.2

Let us have a look at the results for order-based sampling and eight considered samples in comparison with instance-based sampling. For Munich, we achieve superior results for Friday 9, 6, 5 and 1. However, for all other Fridays—both for Munich and Hamburg—less revenue is created. Overall, an increase in revenue of 5.6% and 7.6% can be achieved for Munich and Hamburg, respectively. Compared to the use of instance-based sampling, there is a revenue reduction of 0.7% for Munich and 1.4% for Hamburg. Interestingly, solving more samples hardly changes the results. Even if a higher revenue can be achieved for Munich for some Fridays than when solving fewer samples (Friday 8, Friday 5, Friday 3), a similar or slightly lower revenue is created for most Fridays when solving 20 samples. Overall, the average revenue is 5.5%. For Hamburg, we can also observe individual Fridays where solving 20 samples yields an advantage (Friday 10, Friday 9). For most Fridays, however, there is no advantage of solving more samples.

**Fig. 2:**
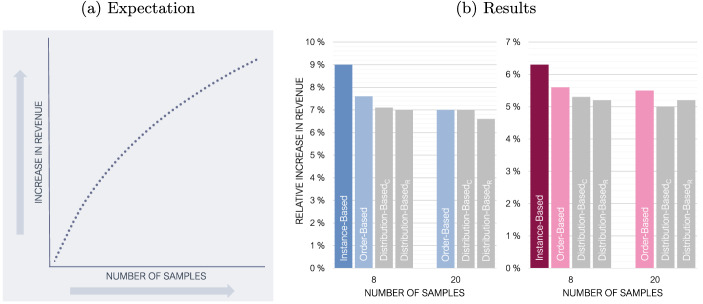
Sampling results for different number of samples in relation to myopic and 8 weeks of data

When we analyze the results of distribution-based sampling, we can see for both Munich and Hamburg that even less revenue can be created (for Munich between 5.0% and 5.2% and for Hamburg between 6.6% and 7.1%). Even if we assume a correlation between the features time window and booking time as observed in the correlation analysis, no noticeable gain can be drawn from this knowledge in the generation of the samples as in distribution-based$_C$ sampling. Only slight differences between solving eight or 20 samples can be found. A combination of the features even leads to worse results here than provided by order-based and instance-based sampling. In Fig. 2, we summarize the results relative to the revenue achieved by myopic. Each figure depicts the revenue collected through sampling relative to myopic on the *y*-axis and the number of samples considered on the *x*-axis as presented in Tables 8 and 9.

**Fig. 3:**
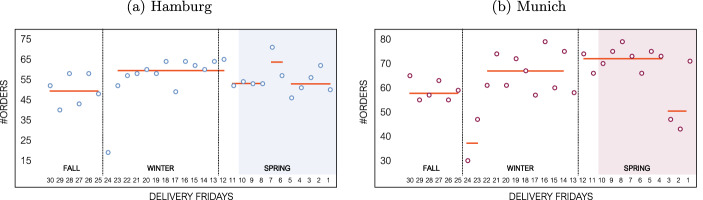
Change point detection in order numbers for all 30 Fridays considered

In addition to revenue, an important selling point for retailers is whether a customer’s time window preference can be fulfilled. Since sampling leads to some customers not being offered certain time windows, we examine how many of the seven total windows are offered to each customer and break these numbers down by customer revenue. We refer to the number of windows offered to customers as service quality.

**Fig. 4:**
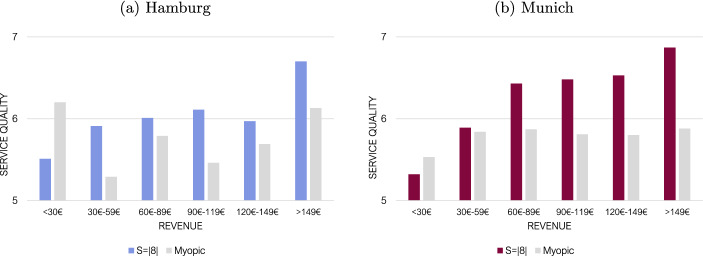
Number of time window options for which a customer’s first-choice can be fulfilled

In Fig. 4a and b, we compare the service quality between the myopic approach and instance-based sampling. The *y*-axis shows the service quality by means of number of offered time windows with seven being the maximum. Along the *x*-axis, the corresponding revenue of the customer is plotted to investigate if the value of the customer has an influence here. In each figure, a colored bar shows the results with $D = S= \vert 8 \vert$, and a gray bar shows the results for the myopic approach. For both cities, it can be seen that the service quality is lower with myopic than with the sampling approach. Furthermore, for both cities, we can see that there is a positive correlation between the service quality and the amount of the revenue—but there is no correlation between revenue and service quality with myopic customer acceptance. Customer requests with a revenue over 149 € have the highest service quality and can choose almost any time window for being accepted. In contrast, for revenues below 30 €, the retailer offers a lower service quality.

*Insights of choosing the best procedure:* We show that it is important to carefully select the features being reflected in the samples. We can discover a clear advantage of order-based and distribution-based sampling over myopic approaches. In contrast to the literature, when considering historical order data, we found that solving more samples does not always lead to better results. This is important because the use of fewer samples may make a sampling-based approach more tractable for online grocers to use in real time.

Next, the more we combine information from the historical order data, the worse the potential requests in the samples seem to represent actually incoming requests. Accordingly, the revenue is lower for order-based and distribution-based sampling than for instance-based sampling. Since order-based and distribution-based require less detailed historical data—namely, only a derivation based on probabilities—they could be of interest for retailers who do not have detailed data or for which a detailed collection of the data is too costly. Also, instance-based sampling can be used to obtain a particularly good fulfillment of first-choice preferences for valuable customers—customers with less value, on the other hand, might perceive a lower quality of service.

## Managerial insights

Our results suggest that data-driven customer acceptance can improve the profitability of retailers significantly by incorporating historical order data. More specifically, our managerial insights are as follows:

- Considering historical order data generally pays off and adds value in a booking process with uncertain demand to identify when capacity should be reserved for potential future requests. Using two different metropolitan regions as case studies, we have shown that we can significantly increase the retailer’s revenue compared to myopic customer acceptance—that has often been implemented by online retailers – by taking historical order data into account.
- It is interesting that considering more historical order data does not necessarily increase the revenue. In our evaluation, in both cities, the highest revenue improvement on average was collected when we considered about two months of historical booking data.
- We have also shown that considering booking data from our case study in the samples as accurately as possible instead of drawing feature values from empirical distributions results in the most additional revenue.
- The previous two findings demonstrate that it is important for online retailers to decide how much and what kind of booking data they collect. Based on our findings, only recent historical data needs to be stored. However, retailers should keep as much detail as possible about their previous booking processes including the first-choice preferences of each customer to know which time windows are most preferred.

## Outlook

Within this work, we presented a data-science procedure to assess the value of historical order data for customer acceptance in attended home deliveries. Our approach is based on a detailed exploratory data analysis, and we believe that by analyzing the data to gain a profound understanding of the problem in a first step, this approach can be applied to any data set of online retailers for attended home deliveries. When considering customer behavior, seasonal or weekday-dependent fluctuations are typical, but have not been included in our analysis. We have based our analysis on the example of Friday deliveries, as in our data and settings, capacity turned out to be particularly tight here. The procedure can be easily transferred to other days or other time windows for capacity management. For practical implementations, computational effort needs to be reasonable, especially when acceptance decisions are made online during the booking process. Although this was not our foremost goal in this study, it turned out that without any further preprocessing of the data, already solving only four samples per request results in higher revenue than greedy acceptance strategies. Furthermore, we have shown that the choice of how much and which historical data to consider has a greater impact on the collected revenue than the number of samples considered.

In the future, we expect that considering historical data will become more important in the area of attended home deliveries. Especially with the ongoing competition in e-commerce and rising customer expectations, considering and anticipating customer behavior from historical data will open new avenues for dynamic pricing and capacity management. Thus, in this context, our work is only a first step toward more systematic evaluation of the value that is added to customer acceptance with historical data. Representing one of the few sampling approaches with real-world data, exploring the value of data for customer acceptance could also be beneficial to the effectiveness of other solution approaches in general.

There is also potential to do more research with the application of order acceptance for grocery deliveries. For example, it would be interesting to see how the techniques presented here would handle data that includes the shift from pre- to post- COVID-19 customer ordering practices. There could be other big shifts, such as a competitor joining or leaving the market, that may require additional pieces to be added to our data understanding or feature combination. However, an unsolved issue is how big shifts in data can be handled. Even without big shifts, it might be useful to examine the value for using real-world data to create additional samples based on historical patterns to use in the sampling-based prediction process. To avoid issues of censored data in sampling and to create a true time-window choice distribution, it is important to store also rejected requests in the retailer database. This could even allow more complex demand management approaches that require detailed choice distributions neglected in this paper.

## Figures and Tables

**Fig. 5:**
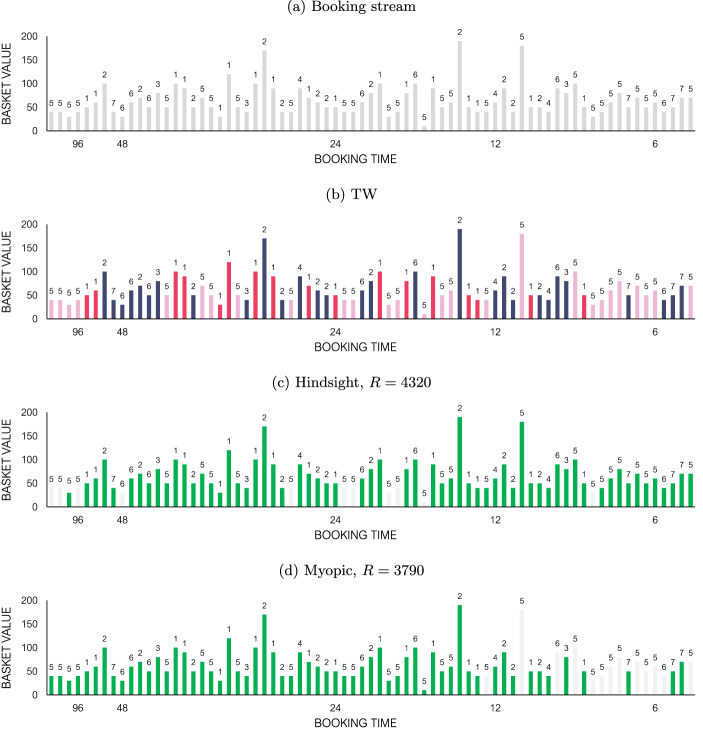
Illustrative example for Friday 7 in Munich

## Data Availability

The data that support the findings of this study were made available to us from the company *AllyouneedFresh*. Therefore, restrictions apply to the availability of these data and so are not publicly available. Data are however available from the corresponding author upon reasonable request.

## Appendix

### Booking fridays

In the following table, we present the characteristics of the 30 booking Fridays taken into account for Hamburg and Munich. The first column indicates how far in the past the Friday is (with a maximum of 30 previous weeks). In column *#Orders* we show how many orders in total were received by AllyouneedFresh on each booking Friday. *Booking time* indicates the average hour before cut-off at which orders were received (hence, the smaller the number the closer orders arrive before cut-off). In *TW*, we show the average TW preferences (with 0 being the first time window option and 7 being the last time window option). *Basket (€)* reflects the average basket value of customers on the respective day. With *Lat., Lon.,* we describe the average coordinates of customers on that day.

See Table 10.

**Table 10:** Friday characteristics for Hamburg and Munich

Friday	Hamburg					Munich				
	#Orders	Booking time	TW	Basket (€)	Lat., Lon.	#Orders	Booking time	TW (€)	Basket	Lat., Long.
30	52	39.46	3.65	63.61	53.571, 9.999	65	27.40	3.77	83.13	48.133, 11.556
29	40	23.55	3.38	55.71	53.587, 10.029	55	16.98	3.94	66.07	48.142, 11.548
28	58	32.02	3.29	62.09	53.567, 10.016	57	21.33	3.55	61.58	48.143, 11.571
27	43	19.19	3.37	61.34	53.569, 10.035	63	33.30	3.64	57.56	48.142, 11.563
26	58	31.33	3.41	67.40	53.567, 10.028	55	30.49	3.73	62.45	48.139, 11.555
25	48	36.21	3.98	72.31	53.560, 10.000	59	28.97	3.54	73.10	48.140, 11.560
24	19	40.00	3.42	71.17	53.578, 10.011	30	33.63	3.58	98.40	48.145, 11.556
23	52	23.67	3.33	74.23	53.552, 10.047	47	24.00	4.07	58.27	48.136, 11.560
22	57	25.54	3.37	62.63	53.569, 10.026	61	18.18	3.32	65.59	48.137, 11.557
21	58	60.66	3.24	72.01	53.566, 10.010	74	53.59	3.57	73.13	48.141, 11.555
20	60	53.92	3.23	62.42	53.573, 10.003	61	37.39	3.91	61.85	48.147, 11.562
19	58	19.81	3.52	75.72	53.570, 10.017	72	24.71	3.23	87.56	48.145, 11.553
18	64	17.70	3.52	57.42	53.571, 10.006	67	20.69	3.83	62.53	48.145, 11.566
17	49	26.39	3.51	65.48	53.567, 10.022	57	17.81	3.86	61.48	48.140, 11.555
16	64	22.78	3.22	64.89	53.571, 10.020	79	22.81	3.53	59.61	48.135, 11.553
15	62	26.35	3.02	62.57	53.577, 10.017	60	25.60	4.02	61.78	48.134, 11.563
14	60	20.32	3.35	54.53	53.571, 10.001	75	26.45	3.46	72.72	48.139, 11.564
13	64	28.86	2.84	65.42	53.564, 10.032	58	32.36	3.49	63.37	48.135, 11.562
12	65	23.12	3.28	66.51	53.552, 10.003	74	22.31	3.69	65.41	48.135, 11.558
11	52	18.21	3.29	58.05	53.577, 10.036	66	26.05	3.24	63.83	48.141, 11.562
10	54	29.31	3.85	66.07	53.582, 10.026	70	32.17	3.86	65.89	48.144, 11.550
9	53	26.87	3.42	65.42	53.580, 10.012	75	33.05	3.58	64.85	48.140, 11.563
8	53	17.62	3.62	68.10	53.573, 10.016	79	22.13	3.72	64.34	48.139, 11.561
7	71	21.25	3.58	63.29	53.570, 10.030	73	27.96	3.67	64.08	48.139, 11.552
6	57	35.93	3.11	61.47	53.568, 10.010	66	33.67	3.47	84.09	48.141, 11.562
5	46	19.20	3.41	67.85	53.561, 10.024	75	22.92	3.44	75.95	48.140, 11.563
4	51	28.67	3.20	70.31	53.575, 10.018	73	35.00	3.18	70.19	48.134, 11.562
3	56	21.98	3.86	63.71	53.585, 10.006	47	17.49	3.55	83.82	48.140, 11.552
2	62	18.68	3.47	64.54	53.566, 10.019	43	21.95	3.42	60.04	48.133, 11.551
1	50	61.46	3.92	66.98	53.572, 10.029	72	39.00	3.54	64.55	48.138, 11.559
Overall ø	54.53	29.00	3.42	64.58	53.570, 10.018	63.60	27.65	3.61	68.12	48.139, 11.558
Overall SD	9.48	11.73	0.25	39.31	0.008, 0.012	11.38	7.74	0.22	68.94	0.004, 0.005

### Benchmark results

We show an illustrative example of how hindsight and myopic handle requests during the booking process, see Fig. 5. Figure 5a visualizes the booking process exactly as observed in historical order data for Friday 7 in Munich. The *x*-axis denotes the arrival time of a request *t* in hours before cut-off. The *y*-axis represents the observed revenue and the chosen time window, indicated with numbers from $0-7$. The first bar on the left represents the first request. Generally, many requests have a revenue of around €50. However, there are three more valuable requests with a revenue of more than €150. In Fig. 5b, we have highlighted the time windows as requested by each customer for the same booking process (TW1 in red, TW5 in pink, and the remaining ones in blue). This demonstrates that most customers within the booking process ask for TW1 and TW5, and that capacity for these two time window is likely to become scarce.

In Fig. 5c and d, the results of hindsight and myopic are shown. Accepted customers are marked in green and rejected customers in gray color. First, hindsight is able to accept 62 customers in total with a total revenue of €4320. Only those customers were rejected that requested TW5 and whose revenue was lower compared to other customers asking for delivery within TW5. Myopic only accepts 60 customers returning an overall revenue of only €3790. Here, all customers were accepted until about $t=12$ hours before the end of the booking process. Then, capacity is becoming too scarce for many of the following requests including one asking for TW5 with a revenue above average.

In summary, first, with very few exceptions, requests that were rejected in hindsight solutions were those that requested the highly demanded time windows TW1 or TW5. This is consistent with the exploratory data analysis and the observed demand for time windows in Fig. 1c and shows that our data set mainly requires capacity management for these two time windows. Second, comparing the solution quality of hindsight and myopic helps us to understand how the booking behavior within each of the Fridays affects the achieved solution quality; it particularly shows the gap in which considering historical order data can have an impact. For the Munich data, on Friday 2 and 3, no capacity management is required, because there is no gap between hindsight and myopic. In contrast, for Hamburg data, on Friday 1 and 7, the gap between myopic and hindsight is particularly large.

### Sampling results with $\theta =1$

To investigate the influence of the chosen acceptance criterion on our results, we additionally investigated a less restrictive criterion in which customers only need to be included in at least one of the sample solutions, i.e., $\theta =1$. Although we can find for all Fridays better results compared to myopic, we could not obtain better results compared to the acceptance limit we use in Sect. 5.3.

See Tables 11 and 12.

**Table 11:** Sampling results in relation to hindsight (H) and myopic (M) in percentages (%) for Hamburg and different horizons *D*

			Friday 10		Friday 9		Friday 8		Friday 7		Friday 6		Friday 5		Friday 4		Friday 3		Friday 2		Friday 1		ø	
			H	M	H	M	H	M	H	M	H	M	H	M	H	M	H	M	H	M	H	M	H	M
Instance-Based	$\vert D \vert = \vert S \vert$	4	**−3.7**	**5.7**	**−1.9**	**3.3**	**−5.4**	**9.8**	**−16.6**	**13.3**	**−6.9**	**9.3**	**−2.7**	**3.6**	−2.1	3.5	**−7.7**	**0.7**	**−8.9**	**−0.6**	**−0.7**	**21.1**	**−5.7**	**7.0**
		8	−4.3	5.0	−1.9	3.3	−5.4	9.8	−17.8	11.7	−8.3	7.7	−3.1	3.3	−3.0	2.5	−7.7	0.7	−6.9	1.5	−4.0	17.0	−6.2	6.2
		12	−7.9	1.0	−4.4	0.7	−6.6	8.4	−18.4	10.9	−11.0	4.5	−3.1	3.3	−3.6	1.9	−7.7	0.7	−6.9	1.5	−4.3	16.6	−7.4	4.9
		16	−6.1	3.0	−4.4	0.7	−13.2	0.7	−24.6	2.4	−11.7	3.6	−3.1	3.3	**−1.2**	**4.4**	−7.7	0.7	−6.9	1.5	−8.3	11.7	−8.7	3.2
		20	−8.8	0.0	−4.4	0.7	−6.6	8.4	−22.3	5.6	−10.0	5.7	−3.1	3.3	−5.4	0.0	−9.2	−1.0	−6.9	1.5	−10.3	9.3	−8.7	3.3

**Table 12:** Sampling results in relation to hindsight (H) and myopic (M) in percentages (%) for Munich and different horizons *D*

			Friday 10		Friday 9		Friday 8		Friday 7		Friday 6		Friday 5		Friday 4		Friday 3		Friday 2		Friday 1		ø	
			H	M	H	M	H	M	H	M	H	M	H	M	H	M	H	M	H	M	H	M	H	M
Instance-Based	$\vert D \vert = \vert S \vert$	4	**−2.8%**	**8.3%**	**−5.2**	**3.0**	−15.2	4.4	**−2.3**	**11.3**	**−4.3**	**7.0**	−8.9	5.5	**−4.4**	**9.1**	**−0.8**	**−0.8**	**0.0**	**0.0**	−4.3	5.0	**−4.8**	**5.3**
		8	−4.4	6.5	−5.5	2.7	−13.8	6.2	−3.0	10.6	−4.3	7.0	−7.8	6.7	−6.6	6.5	−0.8	−0.8	0.0	0.0	−5.0	4.1	−5.1	5.0
		12	−7.0	3.6	−6.1	2.0	**−12.6**	**7.6**	−3.5	10.0	−4.3	7.0	−7.8	6.7	−5.7	7.6	−0.8	−0.8	0.0	0.0	−4.3	5.0	−5.2	4.9
		16	−4.2	6.7	−6.6	1.5	−16.0	3.5	−9.3	3.4	−10.0	0.7	**−10.3**	**3.8**	−5.1	8.3	−0.8	−0.8	0.0	0.0	−4.0	5.2	−6.6	3.2
		20	−4.4	6.5	−6.4	1.7	−16.4	2.9	−9.3	3.4	−10.0	0.7	−10.5	3.6	−8.8	4.0	−0.8	−0.8	0.0	0.0	**−3.8**	**5.5**	−7.0	2.8

## Footnotes

1 www.graphhopper.com.

2 https://cran.r-project.org/web/packages/cpt/cpt.pdf.
